# Temporal Trend of COVID-19 Vaccine Acceptance and Factors Influencing International Travellers

**DOI:** 10.3390/tropicalmed7090223

**Published:** 2022-09-02

**Authors:** Manasvin Onwan, Wasin Matsee, Saranath Lawpoolsri, Phimphan Pisutsan, Tanaya Siripoon, Suda Punrin, Watcharapong Piyaphanee

**Affiliations:** 1 Thai Travel Clinic, Hospital for Tropical Diseases, Faculty of Tropical Medicine, Mahidol University, Bangkok 10400, Thailand; 2 Department of Clinical Tropical Medicine, Faculty of Tropical Medicine, Mahidol University, Bangkok 10400, Thailand; 3 Department of Tropical Hygiene, Faculty of Tropical Medicine, Mahidol University, Bangkok 10400, Thailand; 4 Queen Saovabha Memorial Institute, The Thai Red Cross Society, Bangkok 10330, Thailand

**Keywords:** COVID-19, vaccine acceptance, international traveller, influencing factors, hesitancy, temporal trend

## Abstract

The COVID-19 pandemic has seen disrupted international travel due to travel restrictions and public health measures aimed at containing the spread of the virus. With increasing evidence of the COVID-19 vaccines’ ability to mitigate disease severity, reopening tourism is desirable to promote the recovery of the global economy. However, the COVID-19 vaccine and vaccination passport for international travellers remains an ongoing debate. Little is known of the acceptance of these and the influencing factors among this population group. Therefore, this study sought to determine the temporal trend in COVID-19 vaccine acceptance and influencing factors among international travellers. A cross-sectional study was conducted using a self-administered questionnaire among international travellers who visited the Thai Travel Clinic, Hospital for Tropical Diseases, Mahidol University, Thailand from June 2021 to December 2021 (3 different variants dominated during this period). Study data were analyzed using SPSS software, version 23. Chi-square was used to demonstrate associations. Binary logistic regression was used to evaluate the magnitude of effect, demonstrated by odds ratio with 95% confidence interval. All significant variables were included in a multinomial logistic regression model to estimate adjusted odds ratios. The study enrolled 1068 travellers, 719 (67.3%) Thai and 349 (32.7%) foreign travellers. Most travellers were female (55.4%) and aged 18–30 years. The three main purposes for visiting the clinic were: for study, visiting friends and relatives, and returning to their home country. The overall COVID-19 vaccine acceptance rate among the travellers was 96.2%. The temporal trend of acceptance among Thai and non-Thai travelers varied from 93–99% and 93–100%, respectively. Vaccine efficacy, protective duration of the vaccine, risk of infection, and travel plan were factors strongly associated with COVID-19 vaccine acceptance. In conclusion, the COVID-19 vaccine acceptance rate among these international travellers was very high. The safe and effective reopening of tourism to international travellers will facilitate economic recovery.

## 1. Introduction

The coronavirus disease 2019 (COVID-19) pandemic has impacted individuals, communities, businesses and organisations globally, inadvertently affecting both financial markets and the global economy, including international travel [1]. International travel is among the most significant contributors to COVID-19 spread [2]. Consequently, since the end of 2019, several countries have implemented border control measures and travel restrictions, including pre-departure screening, airport screening and quarantine, to contain the global spread of the virus. The World Tourism Organisation reported that travel destinations worldwide initiated pandemic-related travel restrictions for international tourists, effectively bringing the global travel and tourism sector to a standstill [3]. In light of safe and effective vaccines against COVID-19, many countries hope to reopen tourism and promote recovery of the global economy. Evidence supports that global COVID-19 vaccination is an essential intervention that can greatly reduce disease incidence and severity [4], so tourists can resume travel activity. 

Rapid vaccine development was implemented under Emergency Use Authorisation, which prioritises high-risk populations, including frontline healthcare workers, older adults and those with comorbidities, with the effect of vaccine hesitancy among those eligible for COVID-19 vaccination [5]. Most studies on COVID-19 vaccine acceptance have focused on these high-risk groups, among whom acceptance rates vary. Research results on COVID-19 vaccine acceptance rates among the general population also vary among countries, with the highest vaccine acceptance rates (>90%) in Ecuador (97.0%), Malaysia (94.3%), Indonesia (93.3%) and China (91.3%). In contrast, the lowest vaccine acceptance rates (<60%) were in Kuwait (23.6%), Jordan (28.4%), Italy (53.7%), Russia (54.9%), Poland (56.3%), the United States (56.9%) and France (58.9%) [6]. 

With more vaccine availability and information, international tourism is expected to continue its gradual recovery in 2022, and several countries have lifted their travel restriction to reopen tourism [7]. Currently, Thailand has just opened for international tourists since the first quarter of 2022. Although COVID-19 vaccination is not required to enter Thailand, protocols differ among fully vaccinated, incompletely vaccinated or unvaccinated travellers; for example, incompletely vaccinated and unvaccinated travellers must undergo mandatory quarantine [8]. In this challenging time, little is known about COVID-19 vaccine acceptance and the influencing factors among this special population. Therefore, the aim of this study was to determine the temporal trend of COVID-19 vaccine acceptance and influencing factors among international travellers. We also discuss the acceptance of the vaccine booster dose among international travellers.

## 2. Materials and Methods

### 2.1. Study Design

This study was a cross-sectional, questionnaire-based survey among international travellers who visited the Thai Travel Clinic at the Hospital for Tropical Diseases, Bangkok, Thailand, from June to December 2021. Inclusion criteria were a visit to the clinic as a traveller; age ≥ 18 years; a plan for international travel within 1 month of the clinic visit; the ability to read and understand the Thai or English questionnaire, and willingness to participate in this study. 

**Figure 1:**
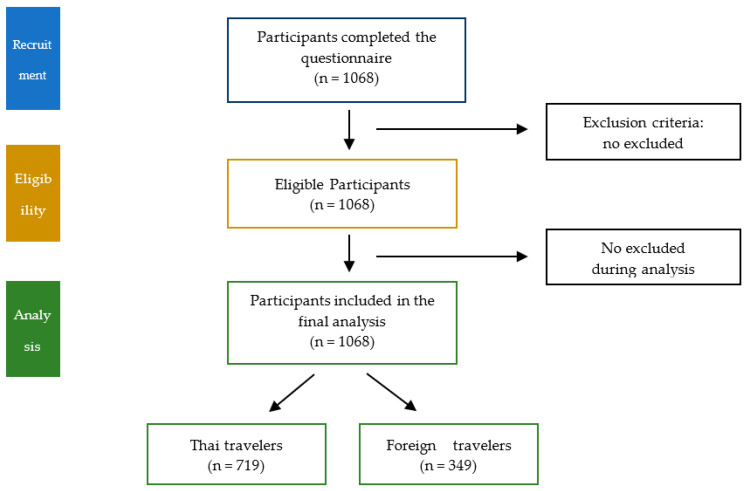
Study flow schematic showing enrolment of 1068 travellers: 719 Thai and 349 foreign (non-Thai) travellers.

The study size was calculated using the following approach to determine the equation for a cross-sectional survey study to estimate the proportion of travellers who accepted the COVID-19 vaccine. Previous studies showed the actual COVID-19 vaccine acceptance rate in the general population was approximately 70% (*p* = 0.7). This study size was calculated with a precision error of 5% and a type I error of 5%; therefore, at least 323 study participants were required. However, because we also intended to observe the temporal trend of vaccine acceptance, a total of 1068 participants were enrolled and included in the final analysis (Figure 1). 

### 2.2. Questionnaire

The paper-based questionnaire consisted of six parts, comprising the primary objectives of (1) demographic data, (2) health status, (3) COVID-19 experience and exposure risk and (4) knowledge and attitude, and the secondary objectives of (5) willingness to receive the vaccine and (6) factors influencing the choice to receive the vaccine. Cronbach Alpha was used to assess the internal consistency of the questionnaire in 30 samples. A score > 0.7 showed high internal consistency and reliability.

Demographic data collected were age, sex, religion, living status, nationality, country of residence, travel destination, purpose of travel, occupation, education level, income and travel clinic visit history. Health status was evaluated using medical conditions, vaccine allergy, smoking history and history of receiving the influenza vaccine in the past 3 years. To evaluate COVID-19 experience and exposure risk, history of COVID-19 testing and infection, including risk in the past year and the next 1 year, was asked. Knowledge about COVID-19 vaccine was evaluated using four questions (yes or no), and the attitude about COVID-19 vaccine was evaluated using four questions (agreed or disagreed). 

The secondary objectives were to determine the travellers’ willingness to receive the COVID-19 vaccine and to identify factors influencing COVID-19 vaccine acceptance, which consisted of vaccine brand, vaccine efficacy, vaccine protective duration, vaccine adverse effects, vaccine price, risk of infection, experience of infection, travel plan and trust in the government. The answers were categorised into four groups (1 = not influenced, 2 = slightly influenced, 3 = influenced and 4 = strongly influenced). Acceptance of COVID-19 vaccination was defined as the willingness to receive the vaccine in terms of the proportion of the study population.

### 2.3. Data Management and Statistical Analysis

Statistical analysis was performed using SPSS software version 23 (IBM, Armonk, NY, USA). Continuous variables were shown as mean and standard deviation or median and interquartile range (IQR), depending on the data distribution; categorical variables were demonstrated as frequency and percentages. Chi-square test was used to demonstrate the association among variables. Binary logistic regression was used to evaluate the magnitude of effect shown using odds ratio (OR) with a 95% confidence interval. A *p*-value < 0.05 was regarded as statistically significant. All significant variables were included in a multinomial logistic regression model to estimate the adjusted ORs.

### 2.4. Ethics

This study was designed in compliance with the Declaration of Helsinki, ICH guidelines for Good Clinical Practice and other International Guidelines for Human Research Protection. This study was approved by the Ethics Committee of the Faculty of Tropical Medicine, Mahidol University (MUTM 2021-030-01). Written informed consent was obtained from all study participants.

## 3. Results

### 3.1. Study Population and Baseline Characteristics

This study enrolled 1068 participants who were classified into two groups: 719 (67.3%) Thai and 349 (32.7%) foreign (non-Thai) participants. Continents represented were Asia for 835 participants, among whom 67.3% were Thai and 10.9% were foreign Asian, in addition to 9.8% from the Americas, 10.6% from Europe, 1.2% from Oceania and 0.2% from Africa. Most of the travel destinations were in North America (41.6%), followed by Europe (30.5%), Asia (21%), Africa (3.4%), Australia and Oceania (2.7%) and South America (0.8%).

The median age was 32 (IQR 26–45) and 36 (IQR 28–50) years among Thai and foreign travellers, respectively. The purpose of travel among Thai participants was studying (31.2%), visiting friends and relatives (18.1%), other (17.8%), tourism (13.2%), returning home (10.6%), business (7.8%) and seminar and meeting conferences (1.3%), whereas the purpose among foreign participants was returning home (33.2%), visiting friends and relatives (26.9%), tourism (16.3%), other (8.9%), business (7.2%), study (7.2%) and seminar and meeting conferences (0.3%). Regarding occupation, most Thai (93.5%) and foreign travellers (93.1%) were non-healthcare workers. For most participants, the reason for visiting the clinic was pre-travel COVID-19 testing (94.3% Thai and 98.6% foreign). Baseline characteristics are shown in Table 1.

### 3.2. Temporal Trends in COVID-19 Vaccine Acceptance

**Figure 2:**
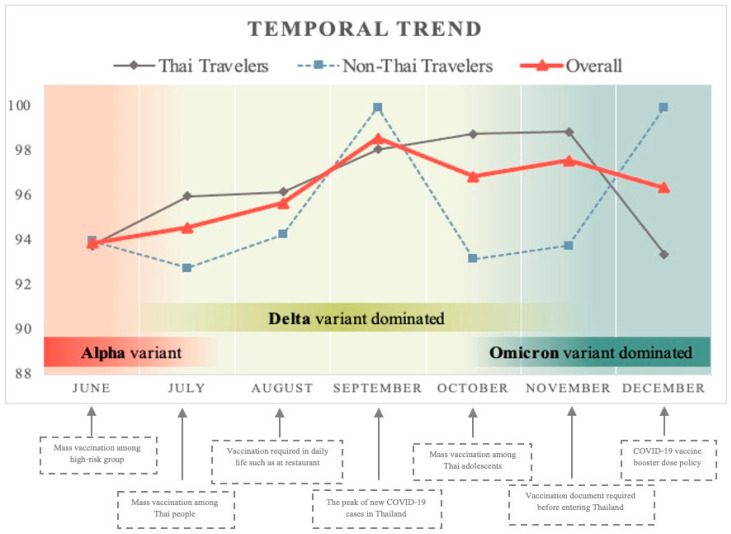
Temporal trends of COVID-19 vaccine acceptance among international travelers from June to December 2021.

The average willingness to obtain the COVID-19 vaccine from June to December 2021 was 96.7% among Thai participants and 95.1% among foreign participants. The overall acceptance rate was 96.2% among all participants. The temporal trends for COVID-19 vaccine acceptance in each month were >90% and varied from 93% to 99% among Thai participants and from 93% to 100% among foreign participants (Figure 2).

### 3.3. Factors Associated with COVID-19 Vaccine Acceptance

**Figure 3:**
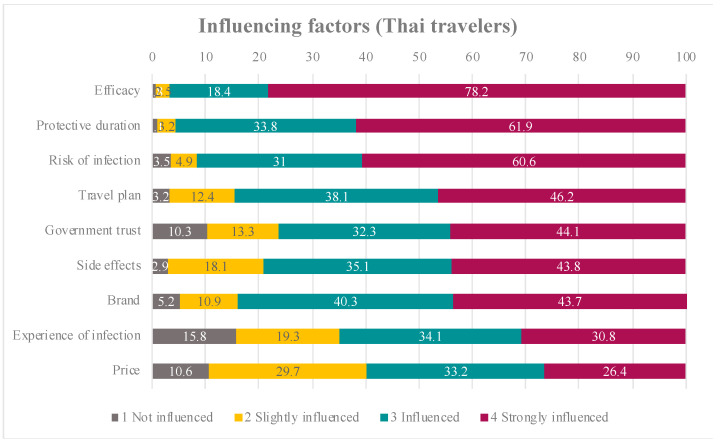
The percentages of influencing factors among Thai travelers.

**Figure 4:**
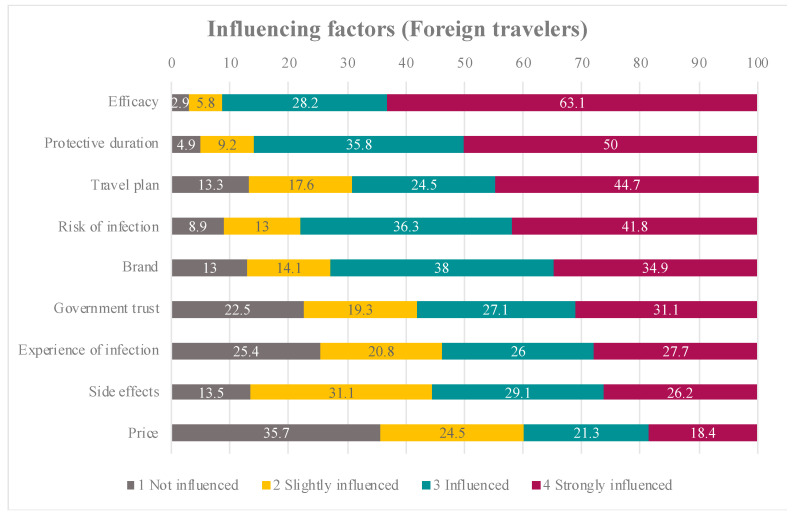
The percentages of influencing factors among foreign (non-Thai) travelers.

Factors influencing COVID-19 vaccine acceptance among Thai and foreign participants are shown in Figure 3 and Figure 4. The top three factors that strongly influenced Thai participants were vaccine efficacy, protective duration of the vaccine and their risk of infection. For foreign participants, vaccine efficacy had the strongest influence, followed by protective duration of the vaccine and their travel plans.

In this study, the acceptance of the COVID-19 vaccine among participants was very high compared with that among those unwilling to receive the vaccine, resulting in an inaccurate and overestimated calculated OR. Therefore, we switched the OR calculation from the acceptance of the COVID-19 vaccine to vaccine hesitancy to find the magnitude of effect, and we interpreted COVID-19 vaccine hesitancy for each factor as shown in Table 2.

Binary logistic regression was used for factor analysis, and the calculated ORs were shown to determine the possible factors for COVID-19 vaccine hesitancy. The cutoff *p*-value was 0.1. The following five factors had a probable association with COVID-19 vaccine hesitancy: (1) at-risk status for COVID-19 over the previous year; (2) a correct answer to the yes or no knowledge question (#1), ‘Vaccine can reduce the severity of the disease’; (3) disagreement with the yes or no attitude questions (#1, #2 and #4), i.e., ‘COVID-19 vaccine should be required before international travel’, ‘The vaccine passport after vaccination would ease you for international travel’ and ‘COVID-19 vaccine will protect you from the disease while travelling’; (4) acceptance of the systemic adverse effects from vaccine; and (5) previous receipt of the COVID-19 vaccine.

Next, the multinomial logistic regression was performed to analyse these factors. It was possible to associate two factors with vaccine hesitancy (*p* < 0.05): (1) travellers who did not recognise that the vaccine can reduce the severity of the disease (adjusted OR 6.00 [1.85–19.49]), and (2) travellers who disagreed that the vaccine passport after vaccination would ease for international travel (adjusted OR 3.24 [1.49–7.04]).

## 4. Discussion

To our best knowledge, this is the first study on COVID-19 vaccine acceptance among international travellers. The temporal trends in the COVID-19 acceptance rate were significantly high among both Thai and foreign travellers. The overall acceptance rate was 96.7% among Thai participants and 95.1% among foreign participants, with a high acceptance rate (>92%) throughout the study period, including 100% acceptance among foreign travellers in September and December 2021. Although the COVID-19 vaccine acceptance or hesitancy has been assessed in various populations [6], the study of international travellers as a special population is limited in terms of being a major contributing factor to COVID-19 transmission. In the light of global hope to resume travel, our findings highlighted a positive sign of recovery for international travel. As an emerging disease, COVID-19 information regarding disease severity, new variants, vaccine development, vaccine effectiveness and vaccine adverse effects has been uncertain and dynamic, with rapid changes over time. Comparison of the vaccine acceptance rate among the study participants is challenging because it may be affected by many factors.

Our study was conducted from June to December 2021, which paralleled the timeline for the three major COVID-19 variants: Alpha, Delta and Omicron. Thailand was hit by the Delta variant as a third wave of the pandemic, with new cases peaking in August, and then the Delta variant remained dominant until the end of November 2021 at the emergence of the Omicron strain. With concern that the Delta variant causes more severe illness than other strains, several studies demonstrated that the individuals infected with the Delta variant were more likely to develop pneumonia and/or require oxygen, and have a significantly increased risk of hospitalisation compared with the wild type or Alpha variant [9,10]. These data increased public fear, resulting in a significantly high vaccine acceptance rate, with a continued high rate during the 7-month study period. In our study, the vaccine acceptance rate was significantly higher than that in a study conducted in Thailand just before the Delta variant became dominant. In that study, conducted in May 2021 among Thai people and expatriates living in Thailand, the acceptance rate was significantly lower compared with that in our study, with an acceptance rate of 57.8% among expatriates and 41.8% among local Thais [11]. Considered together, these findings show that the events of the study period are among the major contributing factors toward the COVID-19 acceptance rate.

Meanwhile, at the beginning of the global vaccine rollout, administration was prioritised for high-risk populations, such as frontline healthcare workers, older adults and individuals with comorbidities. The two major vaccines were CoronaVac, which was imported from China and the AstraZeneca vaccine from the United Kingdom. Despite reports of decreased effectiveness of these vaccines against the Delta variant [12], vaccine acceptance among travellers did not seem impacted.

Our study found that the main purposes for travel during the study period were participating in study-abroad programmes, visiting friends and relatives, and returning to a home country. Although international travellers as a general population with specific purposes were not a priority group for vaccination at the beginning of the rollout, they would come to need the vaccine in the context of becoming high-risk during travel, especially during the period when the Alpha and Delta variants had spread to many countries. Moreover, vaccination status would essentially allow a traveller to attend a university conference, concert and other large events or gatherings without requiring protective measures, such as wearing face masks, social distancing, being in quarantine or testing after arrival. Consequently, some travellers were frustrated by uncertain policies and an increased need for vaccination, contributing to the high acceptance rate in our study.

Previous studies assessed acceptance of other travel vaccines among travellers, such as those for typhoid fever, hepatitis A, influenza and yellow fever, which have an acceptance rate of 60–75% [13,14]. The reasons for the hesitancy were cost and safety concerns, and the perception and awareness of travel-related health problems.

The immunity reached after completing a two-dose COVID-19 vaccination had waned over time [15]. During the Omicron pandemic, the booster dose of the COVID-19 vaccine was needed to improve immunity [16,17]. Although our study did not assess vaccine acceptance for the booster dose, it is worthwhile to emphasise that recommendation of the COVID-19 vaccine booster should be a part of pre-travel consultation amidst the beginning of resuming travel. Further study to investigate acceptance of the COVID-19 vaccine booster doses among travellers should be conducted to provide more information.

Our study demonstrated that factors associated with COVID-19 vaccine acceptance among international travellers were vaccine brand, efficacy, adverse effects and protective duration, in addition to the traveller’s risk of infection and travel plans. Although several studies assessed the factors influencing COVID-19 acceptance [18,19,20,21,22,23,24], our study highlighted the influencing factors among international travellers, which provided different insights from those in previous studies.

Our findings showed that older adult travellers (age > 60 years) totally accepted COVID-19 vaccination. The finding was in contrast to those of a previous study among Thai older adults, which found that 44.3% were hesitant to get the COVID-19 vaccine [23]. Although the COVID-19 vaccine certificate is still not required for international travel due to vaccine equity, according to a World Health Organisation statement [25], having a travel plan was shown to have a positive influence on the willingness to receive the COVID-19 vaccine in 97% of Thai study participants and 87% of foreign participants. This willingness is because vaccination status will exempt vaccinated individuals from some travel restriction measures such as pre-departure testing and quarantine, and will allow them to participate in both indoor and outdoor activities. Moreover, completed vaccination before the trip would enhance the traveller’s confidence to travel safely.

Our study showed that 73.4% of Thai study participants had already received the vaccine, whereas 61% of foreign travellers received the vaccine from their home country before travel. More than 50% of travellers in our study expected vaccine efficacy of more than 70%. Therefore, we demonstrated that travellers’ expectation of vaccine efficacy was strongly influenced by the intention to receive COVID-19 vaccines among travellers. A previous study explained that the relatively high rates of COVID-19 vaccine acceptance may be related to stronger confidence in vaccine effectiveness [6]. Vaccine effectiveness affected the decision by travellers regarding whether to receive the vaccine or not because the high effectiveness led them to trust that vaccination would provide less chance of contracting the disease.

Our study had several limitations. First was the study design, being a convenience sampling in a single travel medicine centre in Thailand where most travellers were well-educated and had an awareness of the need to be vaccinated. Second, participants included a small proportion of foreign travellers due to unexpected travel restrictions and lockdown measures. Third, the study may have several potential biases due to the study design using a questionnaire, which included limited languages. All of these factors may affect generalisation of the results.

## 5. Conclusions

Many factors influenced the intention to receive the COVID-19 vaccine among travellers; however, the acceptance rate among travellers was high compared with that among other groups. It is our hope that the high rate of COVID-19 acceptance among international travellers will allow safe travel and promote recovery of tourism sectors globally.

## Figures and Tables

**Table 1:** Baseline characteristics of this study.

Baseline Characteristics	Thai Travelers *n* = 719 (%)	Foreign Travelers *n* = 349 (%)	*p*-Value
**Age (years)**			
Median (IQR)	32 (26–45)	36 (28–50)	
**Age group**			<0.001
18–30 years	311 (43.3)	102 (29.2)	
31–40 years	161 (22.4)	101 (28.9)	
41–50 years	125 (17.4)	60 (17.2)	
51–60 years	63 (8.8)	41 (11.7)	
>60 years	59 (8.2)	45 (12.9)	
**Gender**			<0.001
Male	255 (35.5)	217 (62.4)	
Female	461 (64.1)	131 (37.5)	
Other	3 (0.4)	0 (0.0)	
Not response	0	1	
**Religion**			<0.001
Buddhism	599 (83.3)	67 (19.2)	
Christianity	34 (4.7)	111 (31.8)	
Islam	15 (2.1)	12 (3.4)	
No religion	59 (8.2)	131 (37.5)	
Other	12 (1.7)	28 (8.0)	
**Living status**			<0.001
Stay alone	171 (23.8)	136 (39.0)	
Couples	147 (20.4)	102 (29.2)	
With family	387 (53.8)	95 (27.2)	
Other	14 (1.9)	16 (4.6)	
**Purpose of travel**			<0.001
Tourism	95 (13.2)	57 (16.3)	
Business	56 (7.8)	25 (7.2)	
Study	224 (31.2)	25 (7.2)	
Seminar and meeting conferences	9 (1.3)	1 (0.3)	
Visiting friends and relatives	130 (18.1)	94 (26.9)	
Returning home	76 (10.6)	116 (33.2)	
Other	128 (17.8)	31 (8.9)	
Not response	1	0	
**Occupation**			0.834
**Healthcare workers**	46 (6.5)	25 (6.9)	
Doctor	7	5	
Dentist	3	0	
Nurse	11	2	
Pharmacist	9	1	
Retired	4	4	
HCW-Other	12	13	
**Non-healthcare workers**	673 (93.5)	324 (93.1)	
Officer	118	32	
Freelance	116	25	
Enterpreneur	31	28	
Seafarer	5	2	
Soldier/Police	9	4	
Diplomat	23	4	
Retired	36	24	
Non-HCW Other	335	205	
**Education level**			0.128
Elementary or lower	10 (1.4)	2 (0.6)	
Secondary	79 (11.3)	39 (11.3)	
Vocational	42 (6.0)	29 (8.4)	
Bachelor	372 (53.0)	160 (46.5)	
Master or higher	199 (28.3)	114 (33.1)	
Not response	17	5	
**Average monthly income**	668	314	<0.001
≤20,000 Baht	208 (31.1)	33 (10.5)	
20,001–50,000 Baht	229 (34.3)	54 (17.2)	
50,001–100,000 Baht	141 (21.1)	87 (27.7)	
100,001–200,000 Baht	59 (8.8)	88 (28.0)	
>200,000 Baht	31 (4.6)	52 (16.6)	
Not response	51	35	
**The purpose of visiting travel clinic**			
Seeking advice	20 (1.9)	0 (0)	0.002
COVID-19 testing before travel	678 (94.3)	344 (98.6)	0.001
Vaccination	48 (6.7)	4 (1.1)	<0.001
Need malaria chemoprophylaxis	1 (1.0)	0 (0)	0.486
Consultation about the illness	0 (0)	0 (0)	-
Other	5 (0.7)	1 (0.3)	0.402
**History of visiting travel clinic before**	136 (18.9)	72 (20.6)	0.507

**Table 2:** Factors associated the vaccine hesitancy.

Factors	Hesitancy *n* (%)		*p*-Value	Crude OR (95% CI)	Adjusted OR * (95% CI)
	Yes	No			
**Traveler type**			0.221		
Thai	24 (3.3)	695 (96.7)		1	
Foreigner	17 (4.9)	332 (95.1)		1.48 (0.79–2.80)	
**Age group**			0.109		
18–30 years	17 (4.1)	396 (95.9)		1	
31–40 years	8 (3.1)	254 (96.9)		0.73 (0.31–1.73)	
41–50 years	9 (4.9)	176 (95.1)		1.19 (0.52–2.72)	
51–60 years	7 (6.7)	97 (93.3)		1.68 (0.68–4.17)	
>60 years	0 (0)	104 (100)		N/A	
**Gender**			0.959		
Male	17 (3.6)	455 (96.4)		1	
Female	24 (4.1)	568 (95.9)		1.13 (0.60–2.13)	
**Religion**			0.341		
Buddhism	21 (3.2)	645 (96.8)		1	
Christianity	9 (6.2)	136 (93.8)		2.03 (0.91–4.54)	
Islam	1 (3.7)	26 (96.3)		1.18 (0.15–9.12)	
No religion	7 (3.7)	183 (96.3)		1.18 (0.49–2.81)	
Other	3 (7.5)	37 (92.5)		2.49 (0.71–8.73)	
**Living status**			0.161		
Stay alone	18 (5.9)	289 (94.1)		1	
Couples	6 (2.4)	243 (97.6)		0.40 (0.16–1.01)	
With family	16 (3.3)	466 (96.7)		0.55 (0.28–1.10)	
Other	1 (3.3)	29 (96.7)		0.55 (0.07–4.30)	
**Purpose of travel**			0.803		
Tourism	7 (4.6)	145 (95.4)		1	
Business	3 (3.7)	78 (96.3)		0.80 (0.20–3.17)	
Study	6 (2.4)	243 (97.6)		0.51 (0.17–1.55)	
Seminar and meeting conferences	1 (10.0)	9 (90.0)		2.30 (0.26–20.79)	
Visiting friends and relatives	10 (4.5)	214 (95.5)		0.97 (0.36–2.60)	
Returning home	7 (3.6)	185 (96.4)		0.78 (0.27–2.29)	
Other	7 (4.4)	152 (95.6)		0.95 (0.33–2.79)	
**Occupation**			0.861		
Healthcare worker (HCW)	3 (4.2)	68 (95.8)		1.11 (0.34–3.70)	
Non-HCW	38 (3.8)	959 (96.2)		1	
**Education level**			0.016		
Elementary or lower	0 (0)	12 (100)		N/A	
Secondary	10 (8.5)	108 (91.5)		1	
Vocational	2 (2.8)	69 (97.2)		0.31 (0.07–1.47)	
Bachelor	23 (4.3)	509 (95.7)		0.49 (0.23–1.06)	
Master or higher	5 (1.6)	308 (98.4)		0.18 (0.06–0.52)	
**Average monthly income**			0.245		
≤20,000 Baht	12 (5.0)	229 (95.0)		1	
20,001–50,000 Baht	13 (4.6)	270 (95.4)		0.92 (0.41–2.05)	
50,001–100,000 Baht	8 (3.5)	220 (96.5)		0.69 (0.28–1.73)	
>100,000 Baht	4 (1.7)	226 (98.3)		0.34 (0.11–1.06)	
**COVID-19 testing before travel**			0.548		
Yes	40 (3.9)	982 (96.1)		1.83 (0.25–13.64)	
No	1 (2.2)	45 (97.8)		1	
**Health status**					
**Underlying diseases**			0.611		
Yes	6 (3.2)	182 (96.8)		1	
No	35 (4.0)	845 (96.0)		1.26 (0.52–3.03)	
**Vaccine allergy**			0.149		
Yes	1 (14.3)	6 (85.7)		4.25 (0.50–36.17)	
No	40 (3.8)	1021 (96.2)		1	
**Smoking**			0.192		
Yes	5 (8.2)	56 (91.8)		2.41 (0.90–6.42)	
Ex-smoker	4 (3.7)	104 (96.3)		1.04 (0.36–3.00)	
No	32 (3.6)	863 (96.4)		1	
**History of receiving influenza vaccination in the past 3 years**			0.090		
Never	27 (5.0)	508 (95.0)		1	
1 time	9 (3.6)	244 (96.4)		0.69 (0.32–1.50)	
2 times	3 (2.2)	133 (97.8)		0.42 (0.13–1.42)	
3 times	1 (0.8)	128 (99.2)		0.15 (0.02–1.10)	
**COVID-19 experiences and exposure**					
**Diagnosed with COVID-19 before**			0.258		
Yes	0 (0)	31 (100)		N/A	
No	41 (4.0)	994 (96.0)			
**Friends and family or relatives have been diagnosed with COVID-19**			0.825		
Yes	7 (4.1)	162 (95.9)		1.10 (0.48–2.52)	
No	34 (3.8)	864 (96.2)		1	
**At risk of getting COVID-19 over the past year**			0.027		*p*-value = 0.14
Yes	4 (1.8)	215 (98.2)		1	1
No	34 (5.1)	639 (94.9)		2.86 (1.00–8.15)	2.26 (0.77–6.64)
**At risk of getting COVID-19 in the next 1 year**			0.491		
Yes	8 (3.4)	224 (96.6)		1	
No	21 (4.6)	431 (95.4)		1.36 (0.60–3.13)	
**Knowledge**					
**Q1: Vaccine can reduce the severity of the disease**			<0.001		*p*-value < 0.01
Correct	34 (3.3)	1008 (96.7)		1	1
Incorrect	7 (31.8)	15 (68.2)		13.84 (5.30–36.14)	6.00 (1.85–19.49)
**Q2: Vaccine can give 100% protection from the disease**			0.074		
Correct	41 (4.1)	949 (95.9)		-	
Incorrect	0 (0)	74 (100)		-	
**Q3: After vaccination, vaccine can protect you from the disease immediately**			0.598		
Correct	35 (4.0)	838 (96.0)		1.27 (0.53–3.06)	
Incorrect	6 (3.2)	182 (96.8)		1	
**Q4: After vaccination, wearing a mask is not necessary**			0.514		
Correct	40 (3.9)	975 (96.1)		1.93 (0.26–14.33)	
Incorrect	1 (2.1)	47 (97.9)		1	
**Knowledge** **(Total scores)**			0.173		
All correct (4 scores)	28 (3.4)	785 (96.6)		1	
Any incorrect (1–3 scores)	13 (5.4)	229 (94.6)		1.59 (0.81–3.12)	
**Attitude**					
**Att1: COVID-19 vaccine should be required before international travel**			<0.001		*p*-value = 0.14
Agree	22 (2.5)	855 (97.5)		1	1
Disagree	19 (10.1)	169 (89.9)		4.37 (2.31–8.25)	1.86 (0.82–4.12)
**Att2: The vaccine passport after vaccination would ease you for international travel**			<0.001		*p*-value < 0.01
Agree	26 (2.8)	919 (97.2)		1	
Disagree	15 (12.4)	106 (87.6)		5.00 (2.57–9.71)	3.24 (1.49–7.04)
**Att3: After completed COVID-19 vaccination, quarantine is not necessary after international travel**			0.910		
Agree	26 (3.8)	655 (96.2)		1	
Disagree	15 (4.0)	364 (96.0)		1.04 (0.54–1.99)	
**Att4: COVID-19 vaccine will protect you from the disease while traveling**			0.006		*p*-value = 0.17
Agree	21 (2.8)	726 (97.2)		1	1
Disagree	20 (6.3)	295 (93.7)		2.34 (1.25–4.39)	1.62 (0.81–3.24)
**The least expectation toward COVID-19 vaccine efficacy**			<0.001		
50–59%	4 (4.1)	93 (95.9)		1	
60–69%	2 (2.5)	79 (97.5)		0.59 (0.11–3.30)	
70–79%	2 (0.9)	216 (99.1)		0.22 (0.04–1.20)	
80–89%	8 (3.3)	236 (96.7)		0.79 (0.23–2.68)	
≥90%	9 (4.9)	176 (95.1)		1.19 (0.36–3.96)	
No matter how much efficacy is, I will receive vaccine	8 (3.4)	224 (96.6)		0.83 (0.24–2.83)	
No matter how much efficacy is, I will NOT receive vaccine	8 (100)	0 (0)		N/A	
**The least acceptable protective duration of COVID-19 vaccine**			0.002		
<6 months	15 (5.3)	267 (94.7)		1	
6–11 months	8 (2.1)	369 (97.9)		0.39 (0.16–0.92)	
1–2 years	10 (3.1)	314 (96.9)		0.57 (0.25–1.28)	
>2 years	8 (10.7)	67 (89.3)		2.13 (0.87–5.22)	
**Acceptable side effect(s) of the vaccine**					
**Local side effects**			0.191		
Yes	31 (3.4)	872 (96.6)		1	
No	9 (5.6)	153 (94.4)		1.66 (0.77–3.54)	
**Systemic side effects**			0.025		*p*-value = 0.26
Yes	17 (2.7)	617 (97.3)		1	1
No	23 (5.3)	408 (94.7)		2.05 (1.08–3.88)	1.48 (0.75–2.95)
**Severe allergy or anaphylaxis**			0.014		
Yes	3 (13.6)	19 (86.4)		1	
No	37 (3.6)	1005 (96.4)		0.23 (0.07–0.82)	
**Death**			0.037		
Yes	2 (14.3)	12 (85.7)		1	
No	38 (3.6)	1013 (96.4)		0.23 (0.05–1.04)	
**The highest price that you are willing to pay**			0.256		
≤1000 Baht	25 (5.0)	479 (95.0)		1	
1001–2000 Baht	6 (2.6)	223 (97.4)		0.52 (0.21–1.27)	
2001–3000 Baht	3 (2.0)	146 (98.0)		0.39 (0.12–1.32)	
3001–4000 Baht	2 (3.1)	63 (96.9)		0.61 (0.14–2.63)	
4001–5000 Baht	0 (0)	29 (100)		N/A	
>5000 Baht	1 (1.4)	68 (98.6)		0.28 (0.04–2.11)	
**You have received COVID-19 vaccine before**			0.004		*p*-value = 0.17
Yes	20 (2.7)	719 (97.3)		1	1
No	21 (6.4)	306 (93.6)		2.47 (1.32–4.62)	1.68 (0.80–3.55)

* Adjusted OR by the following factors (*p*-value < 0.1); at risk of getting COVID-19 over the past year, knowledge question no.1, attitude question no.1,2,4, accepted systemic side effect, previous receive COVID-19 vaccine before.

## Data Availability

The data for this study has been presented within this article and any further information regarding this study can be reasonably requested from the corresponding author.
